# High-Intensity Interval Training Improves Physical Function, Prevents Muscle Loss, and Modulates Macrophage-Mediated Inflammation in Skeletal Muscle of Cerebral Ischemic Mice

**DOI:** 10.1155/2021/1849428

**Published:** 2021-11-20

**Authors:** Lu Luo, Meixi Liu, Hongyu Xie, Yunhui Fan, Jingjun Zhang, Li Liu, Yun Li, Qiqi Zhang, Junfa Wu, Congyu Jiang, Yi Wu

**Affiliations:** Department of Rehabilitation Medicine, Huashan Hospital, Fudan University, Shanghai, China

## Abstract

Although skeletal muscle is the main effector organ largely accounting for disability after stroke, considerably less attention is paid to the secondary abnormalities of stroke-related skeletal muscle loss. It is necessary to explore the mechanism of muscle atrophy after stroke and further develop effective rehabilitation strategy. Here, we evaluated the effects of high-intensity interval (HIIT) versus moderate-intensity aerobic training (MOD) on physical function, muscle mass, and stroke-related gene expression profile of skeletal muscle. After the model of middle cerebral artery occlusion (MCAO) was successfully made, the blood lactate threshold corresponding speed (*S*_LT_) and maximum speed (*S*_max_) were measured. Different intensity training protocols (MOD < *S*_LT_; *S*_LT_ < HIIT < *S*_max_) were carried out for 3 weeks beginning at 7 days after MCAO in the MOD and HIIT groups, respectively. We found that both HIIT and MOD prevented stroke-related gastrocnemius muscle mass loss in MCAO mice. HIIT was more beneficial than MOD for improvements in muscle strength, motor coordination, walking competency, and cardiorespiratory fitness. Furthermore, HIIT was superior to MOD in terms of reducing lipid accumulation, levels of IL-1*β* and IL-6 in paretic gastrocnemius, and improving peripheral blood CD4+/CD8+ T cell ratio, level of IL-10. Additionally, RNA-seq analysis revealed that the differentially expressed genes among HIIT, MOD, and MCAO groups were highly associated with signaling pathways involved in inflammatory response, more specifically the I-kappaB kinase/NF-kappaB signaling. Following the outcome, we further investigated the infiltrating immune cells abundant in paretic muscles. The results showed that HIIT modulated macrophage activation by downregulating CD86+ (M1 type) macrophages and upregulating CD163+ (M2 type) macrophages via inhibiting the TLR4/MyD88/NF*κ*B signaling pathway and exerting an anti-inflammatory effect in paretic skeletal muscle. It is expected that these data will provide novel insights into the mechanisms and potential targets underlying muscle wasting in stroke.

## 1. Introduction

Stroke is one of the leading causes of disability worldwide and imposes a tremendous burden on victims, families, and healthcare systems [1]. About 50% of stroke patients have hemiplegia, and 30% of them were unable to walk without assistance [2]. Although the skeletal muscle is the main effector organ largely accounting for disability in stroke patients, most of the researches on motor dysfunction after stroke focus on the concept of neurovascular unit throughout the last two decades [3, 4]. Considerably less attention is paid to the secondary abnormalities of stroke-related skeletal muscle loss or sarcopenia.

Sarcopenia is defined as “progressive and comprehensive syndrome of skeletal muscle loss and strength decline” with increased risk of adverse consequences including higher mortality, quality of life decline, increased rate of falls, and fractures [5]. Sarcopenia is divided into primary sarcopenia caused by aging, and secondary sarcopenia which is activity-related, nutrition-related, or disease-related [6]. Stroke-related sarcopenia is independent of age, showing a rapid muscle mass loss, and significantly bilateral differences in physical and functional performance [7]. The prevalence of sarcopenia can reach 15% in healthy elderly and 56% in rehabilitated patients. The most widely recognized etiologic factors include denervation, insulin resistance, poor nutritional status, physical inactivity, and inflammation [8].

Despite that sarcopenia contributes to disability and negative outcome after stroke, current clinical practice guideline recommendations fail to adequately address the peripheral muscle adaptations poststroke [9]. Currently, treatment for patients with sarcopenia includes nutritional supplements and hormone-related treatments to improve nutritional status, and weight, which may cause fluid retention, hypogonadism, and orthostatic hypotension [10]. Emerging data suggest that exercise or resistance training is considered the most effective strategy currently available for increasing muscle mass and strength and improving physical function [11, 12]. However, essential training time and sufficient exercise intensity are lacking in most traditional stroke rehabilitation programs [13]. Studies have shown that 76% of time was spent in bed or sitting in the hospitalized stroke, and only 23% of their time are standing or walking [14]. In healthy elderly people, muscle protein synthesis will reduce 30% and muscle mass in lower limbs will reduce 6% after 10 days of bed rest, resulting in a 16% reduction in muscle strength [15]. Well-controlled clinical trials involving the health and patients with stroke have confirmed that high-intensity interval training (HIIT), a new strategy consisting of alternating periods of greater and lower intensity within an exercise session, is more significantly effective and safe than moderate-intensity training for improvements in aerobic capacity, insulin sensitivity, and mitochondrial function [16, 17]. However, the effect of HIIT on physical or muscle mass and composition remains controversial. There is no conclusive evidence to prescribe a specific exercise program in terms of type, intensity, frequency, and duration. Further mechanistic studies are required to develop more effective strategies for preventing or reversing muscle wasting and improving rehabilitation success in patients or animal models with stroke. Therefore, the objective of the present study was to evaluate the effects of high-intensity interval versus moderate-intensity aerobic training on physical function, skeletal muscles, and molecular changes at the genome level in cerebral ischemic mice.

## 2. Materials and Methods

### 2.1. Animals

Male C57BL/6 mice (20-25 g), purchased from Shanghai Jihui Laboratory Animal Care Co., Ltd., were initially housed in standard plastic cages (cage size: 26 × 19 × 15 cm) in a temperature-controlled environment (22 ± 2°C) with 50 ± 10% humidity under a 12/12 h light/dark cycle (lights on 7:00 a.m.) with sufficient food and water. All experiment procedures were performed according to the National Institutes of Health guide for the Care and Use of Laboratory Animals. Before use, the mice were allowed to acclimate to laboratory conditions for seven days.

### 2.2. Middle Cerebral Artery Occlusion Surgery

The middle cerebral artery occlusion (MCAO) model of left middle cerebral artery ischemia for 60 minutes was established after anesthesia by 1% pentobarbital sodium (10mg/kg. ip). The blood flow of the middle cerebral artery was monitored by a laser speckle blood flow meter (RWD Life Science, Shenzhen, China). TTC staining was used to identify the area of infarction. The neurological deficit level of the mice was examined using the modified neurological severity score (mNSS) 24 hours after MCAO surgery. MCAO mice with mNSS more than 6 participated in the experiment (see the Supplementary material) (available here).

After MCAO, mice were excluded according to the following criteria: (1) mNSS less than 6 or overactive (*n* = 17); (2) resisting running on a treadmill (*n* = 5); (3) death during or after the surgery and ineffective arterial occlusion (*n* = 12). Overall, 80 mice were used, and 46 of them were included. Mice were randomly assigned to the sham group (*n* = 10), MCAO group (*n* = 12), MOD group (*n* = 12), and HIIT group (*n* = 12).

### 2.3. Incremental Test and Exercise Protocols

The protocol was performed with the following adaptations according to the previously published work [18]. Seven days after MCAO, the mice in HIIT and MOD groups were placed on the motor-driven treadmill (Huaibei Zhenghua Biological Instrument Equipment Co. Ltd, China) and warmed up at a rate of 6 m/min for 5 min. Then, accelerate by 3 m/min every 3 minutes until the mouse cannot maintain the applied speed, and the final speed is defined as the maximum speed (*S*_max_). Every 20 seconds of acceleration, a small amount of blood (0.2 *μ*L) was collected from the tail vein. The blood lactate concentration (mmol/L) was measured by a portable blood lactate device (Lactate Scout+, EKF Diagnostics, Germany). When the blood lactate concentration measured twice in a row has a significant inflection point or is increased by 1 mmol/L, the blood lactate concentration measured in the previous measurement is regarded as the lactate threshold (LT), and the corresponding treadmill speed is called *S*_LT_.

HIIT: the session consists of a 4 × 4-minute high-intensity treadmill run (*S*_LT_ + 60‐70%(*S*_max_ − *S*_LT_)), interrupting active recovery (*S*_LT_) for 3 minutes between each intensity series. The plan is implemented five times a week for three weeks.

MOD: the speed is fixed at 80-90% *S*_LT_ to avoid the accumulation of lactate. The project is carried out every day for 3 consecutive weeks. In order to match the total energy expenditure (*W*) between the groups and only compare the effects of intensity, according to the energy expenditure (exercise + recovery) of the HIIT group, the daily exercise time of the MOD group was adjusted by the following formula:

(1) $$\begin{array}{c} W\;\left(\text{J}/\text{kg}\cdot \text{m}\right)=\text{mass }\left(\text{kg}\right)\times \text{speed }\left(\text{m}/\min\right)\times \text{time }\left(\min\right)\times \text{treadmill tilt }\left({}^{{}^{\circ}}\right)\times 9.8. \end{array}$$

Each program included a 5-minute warm-up (50% *S*_LT_) before the formal training. The *S*_max_ and *S*_LT_ of the mice were retested every two weeks to adjust the training intensity. The mice in the sham sedentary group were housed in a crowded cage (cage size: 26 × 19 × 15 cm, 8/per cage) with no access to the treadmill.

### 2.4. Behavioral Tests

The behavioral tests were performed 2 days before and 3 weeks after exercise intervention. The test sequence was as follows: (1) open field test, (2) grip strength test, (3) rotarod test, (4) cylinder test, (5) ladder rung walking test, and (6) CatWalk XT gait test (see the Supplementary material).

### 2.5. Pulmonary Function

The pulmonary function of mice was evaluated by a whole-body plethysmograph. Briefly, three days before the test, mice were placed in a sealed box that was connected to transducers and a computer and allowed to acclimate for 15 min every day. On the test day, the mice were placed again in the box to acclimate for 5 min. Afterwards, pulmonary function was recorded and assessed for 5 min, including peak expiratory flow (PEF), peak inspiratory flow (PIF), and minute volume (MV) and tidal volume (TV).

### 2.6. Body Weight and Muscle Mass

The body weight of the mice was measured at fixed times every 4 days until the end of the experiment, and the body weight growth rate was calculated as follows: growth rate (%) = (current body weight − body weight at the first day)/body weight at the first day × 100%. Paretic gastrocnemius muscle of mice in four groups was isolated, removed, and weighed after being deeply anesthetized.

### 2.7. Experimental Material Preparation

Mice were deeply anesthetized by 1% pentobarbital sodium (10 mg/kg. ip). For western blotting, RNA sequencing, adenosine triphosphate (ATP)-ase staining, and Oil-Red-O staining, mice in each group were sacrificed and paretic gastrocnemius was quickly removed, placed in Eppendorf tubes, frozen in liquid nitrogen, and stored at -80°C for further use. For immunohistochemistry, the mice were transcardially perfused with 50 mL of phosphate-buffered saline (PBS) and then fixed with 50 mL 4% paraformaldehyde (PFA) solution. The paretic gastrocnemius was removed, postfixed for 24 h in the same fixative, and cryoprotected 24 h at 4°C in a 30% sucrose solution. After then, the tissue blocks were embedded in paraffin for further use. Blood sample (300 *μ*L) was collected by enucleating the mouse eyeball for flow cytometric analysis.

### 2.8. Hematoxylin and Eosin (HE) Staining and Immunohistochemistry

The serial coronal sections in the maximum cross section of paretic gastrocnemius were made to observe the morphology of muscle cells, infiltrating cells by HE staining, and to detect the distribution of CD86 and CD163 proteins by immunohistochemistry (see the Supplementary Materials).

### 2.9. Adenosine Triphosphate (ATP)-ase Staining and Oil-Red-O Staining

The transverse serial sections were incubated with calcium chloride solution for 5 min and calcium chloride solution for 30 min. Then, the sections were stained with calcium chloride, cobalt nitrate, and ammonium sulphide solutions. Type I muscle fiber is light gray or colorless, and type II muscle fiber is dark gray or black. For Oil-Red-O (ORO) staining to detect lipid deposition, slides were immersed after washing with PBS for 15 min in the ORO working solution and rinsed with deionized water.

### 2.10. RNA Sequencing and Differentially Expressed Gene Analysis

Total RNA was extracted using the TRIzol reagent according to the manufacturer's protocol. GO enrichment and KEGG pathway enrichment analysis of differentially expressed genes (DEGs) were performed respectively using R based on the hypergeometric distribution. The transcriptome sequencing and analysis were conducted by OE Biotech Co., Ltd. (Shanghai, China) (see the Supplementary Materials).

### 2.11. Flow Cytometry

To determine the percentage of total leukocytes and lymphocyte subsets, blood samples were stained with various monoclonal antibodies (mAbs) and evaluated by flow cytometry. Cells were analyzed on Becton-Dickinson FACSCalibur flow cytometer using FlowJo software (see the Supplementary Materials).

### 2.12. Profiling of Cytokines/Chemokines

Cytokines and chemokines in paretic gastrocnemius were measured and quantified using the LEGENDplex™ mouse inflammation panel (BioLegend, 740446) according to the manufacturer's instructions. LEGENDplex™ software was used for analyzing collected data (BioLegend) (see the Supplementary Materials).

### 2.13. Western Blot Assays

The expression of CD86, CD163, TLR4, MyD88, NF*κ*B, and p-NF*κ*B proteins in the paretic gastrocnemius was detected by western blotting after 3 weeks of training (see the Supplementary Materials).

### 2.14. Statistical Analysis

Data are expressed as the mean ± standard error (SEM) of at least three independent experiments.

Statistical tests were done on SPSS 23.0 statistical software (SPSS, Chicago, IL, USA) and GraphPad Prism 9.0 (GraphPad Software Inc., USA). Firstly, a normality test was performed. One-way analysis of variance (ANOVA) for multiple comparisons followed by Tukey's post hoc test was performed for the data with normal distribution. The Kruskal–Wallis test was performed for the data with nonnormal distribution. Statistical significance between two groups was determined with unpaired Student's *t*-test. A probability of 0.05 or less was considered statistically significant.

## 3. Results

### 3.1. Establishment of the Animal Model of Cerebral Ischemia Which Induced Skeletal Muscle Atrophy

**Figure 1:**
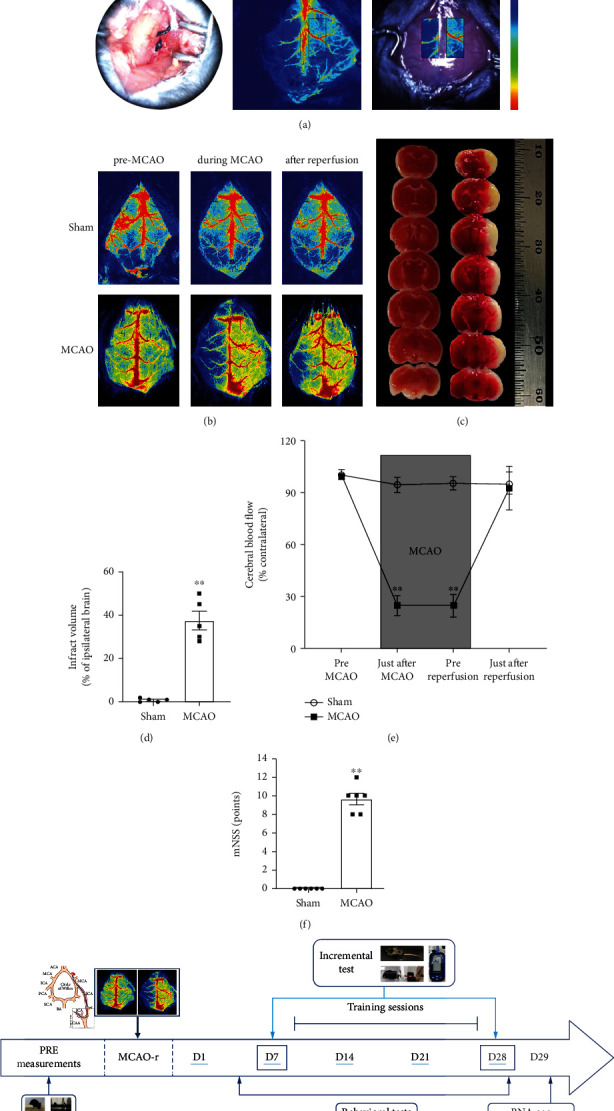
Establishment of the animal model of cerebral ischemia. (a) Middle cerebral artery occlusion (MCAO) was established under cerebral blood flow monitored by laser speckle blood flow meter. (b) Pseudocolor picture of cerebral blood flow before, during, and after MCAO. (c) Triphenyl tetrazolium chloride (TTC) staining showed the infarct size after cerebral ischemia reperfusion. (d) Quantification of cerebral infarct volume of the ipsilateral brain between sham and MCAO groups (*n* = 6). (e) Quantification of cerebral blood flow in the ipsilateral hemisphere normalized to the contralateral hemisphere. (f) Modified neurological severity score (mNSS) of the sham-operated and ischemic mice (*n* = 6). (g) Experimental design of the study. Mice were subjected to MCAO after baseline assessments. Then, animals were randomly subdivided into sham, sedentary MCAO, moderate-intensity aerobic training (MOD), and high-intensity interval training (HIIT) groups 1 week after MCAO. Following 3 weeks of isocaloric training sessions (MOD and HIIT) or sedentarism (sham and MCAO), all groups underwent posttraining assessments. Values are expressed as the mean ± SEM of the mean. ^∗^*P* < 0.05 and ^∗∗^*P* < 0.01 as determined by unpaired *t*-test.

**Figure 2:**
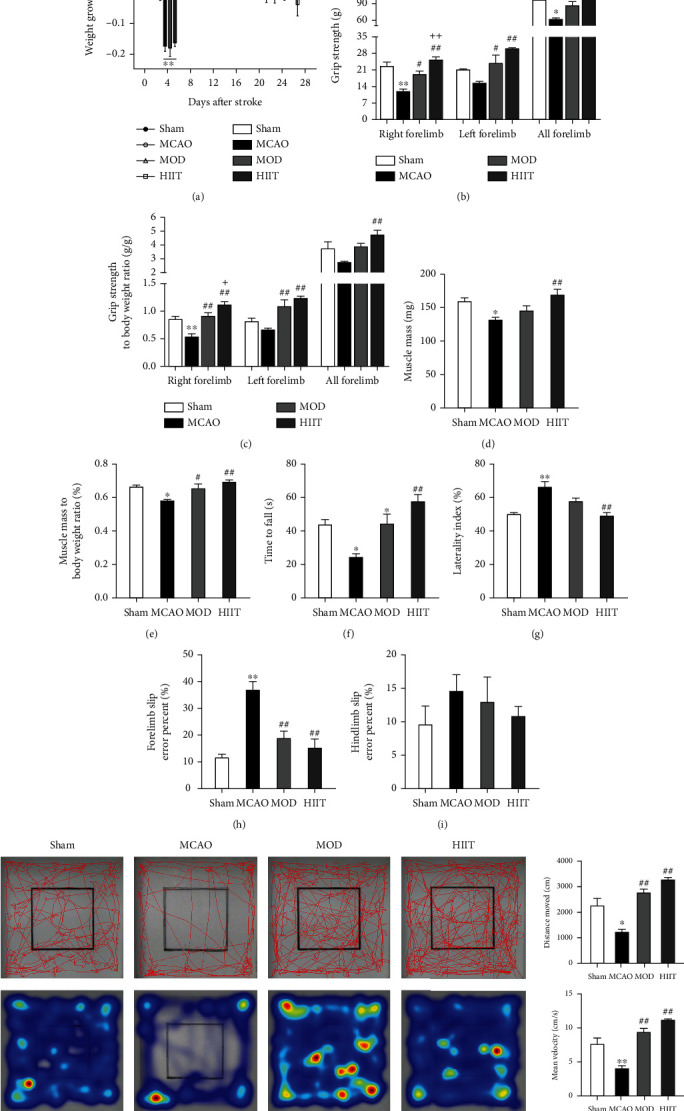
Amelioration of muscle wasting and motor dysfunction after exercise protocols. (a) Changes in mean values of total body weight and weight growth rate (data are expressed as the change with respect to day 0) in each group. (b) The grip strength (g) of bilateral forelimbs measured using a digital force gauge in each group (*n* = 5). (c) The ratio of grip strength and body weight in bilateral forelimbs in each group (*n* = 5). (d) Muscle mass (mg) of paretic gastrocnemius in each group (*n* = 6). (e) The ratio of paretic gastrocnemius mass and body weight in all groups (*n* = 6). (f) Time course of the latency to fall off the rotarod in the rotarod test in each group (*n* = 6). (g) The percentage of laterality in the cylinder test in each group (*n* = 6). (h, i) The percent of forelimb slips and hindlimb slips in the ladder rung walking test in each group (*n* = 6). (j) Motion trajectory and trajectory endpoint heat map of each mouse in the open field test (left); the total distance of motion trajectory (cm) and mean velocity (cm/s) of each mouse (right) (*n* = 6). Values are expressed as the mean ± SEM of the mean. ^∗^*P* < 0.05 and ^∗∗^*P* < 0.01 compared with the sham group; ^#^*P* < 0.05 and ^##^*P* < 0.01 compared with the MCAO group; ^+^*P* < 0.05 and ^++^*P* < 0.01 compared with the MOD group as determined by one-way ANOVA (Tukey's multiple comparison test) for the data with normal distribution. The letters for no significance were not shown.

To determine the change of morphology and function of poststroke skeletal muscle, a cerebral ischemic animal model was established by the MCAO method, which significantly caused ~40% ipsilateral brain infarcts in the lateral striatum and cortex regions shown by TTC staining (*P* < 0.01; Figures 1(c) and 1(d)). The ischemic cerebral blood flow dropped below 30% of the contralateral side during surgery monitored by laser speckle blood flow meter (*P* < 0.01; Figures 1(b) and 1(e)), which severely induced motor deficit as evidenced by higher neurological score (*P* < 0.01; Figure 1(f)) and lower distance moved (*P* < 0.05; Figure 2(j)). Total body weight of ischemic mice was rapidly decreased and significantly lower compared to sham-operated mice. Weight loss peaked at day 4 and recovery of body weight (starting at day 4) remained incomplete and slightly lower compared to the sham group until the end of the study (Figure 2(a)). Muscle mass loss was observed in gastrocnemius muscle of the contralateral leg in the 4th week poststroke. Next, we calculated the ratio of gastrocnemius mass to body weight; the MCAO group also showed a significant decrease in the ratio relative to the sham group (*P* < 0.05; Figures 2(d) and 2(e)).

### 3.2. Exercise Protocols of High-Intensity Interval Training and Moderate-Intensity Aerobic Training

**Figure 3:**
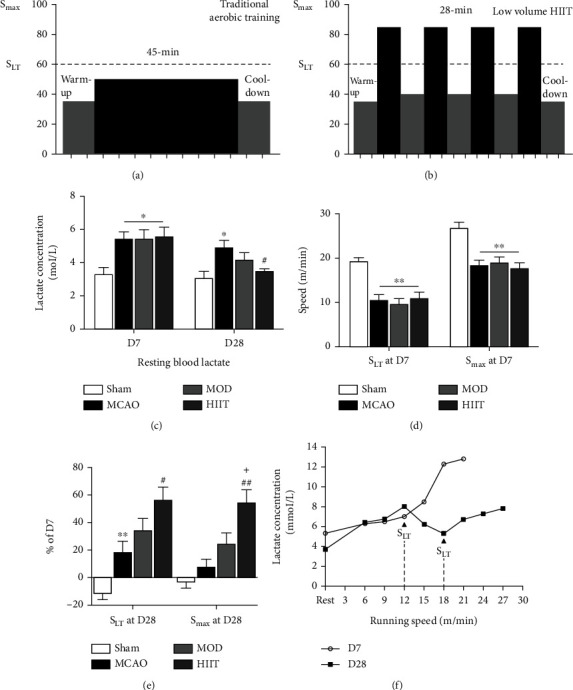
Exercise protocols of high-intensity interval training and moderate-intensity aerobic training. (a, b) Regimens of traditional moderate-intensity aerobic training and low volume high-intensity interval training. (c) Relative percentage (%) of resting blood lactate (mmol/L) at D28 normalized to the D1 (*n* = 6). (d) *S*_LT_ and *S*_max_ (m/min) at D7 in each group (*n* = 6). (e) Relative percentage (%) of *S*_LT_ and *S*_max_ at D28 normalized to the D1 (*n* = 6). (f) Example of lactatemia kinetic (raw data) before and after HIIT. Arrows indicate the lactate threshold. Values are expressed as the mean ± SEM of the mean. ^∗^*P* < 0.05 and ^∗∗^*P* < 0.01 compared with the sham group; ^#^*P* < 0.05 and ^##^*P* < 0.01 compared with the MCAO group as determined by one-way ANOVA (Tukey's multiple comparison test) for the data with normal distribution. The letters for no significance were not shown. Abbreviation: *S*_LT_: speed associated with the lactate threshold; *S*_max_: maximal speed; D7: 7 days after cerebral ischemia; D28: 28 days after cerebral ischemia.

Different from the moderate-intensity aerobic training recommended by some stroke rehabilitation guidelines [9], we measured the blood lactate threshold (LT) that most MCAO mice could reach during exercise to quantify high-intensity training and low-to-moderate-intensity aerobic training (Figures 3(a) and 3(b)). The intensity below LT is located in the ranges of the moderate intensity (e.g., 40-70% of VO_2_ peak). The resting blood lactate concentration of the sedentary MCAO mice was higher than that in the sham group at the 7th day (D7) and was reversed by 3 weeks of HIIT at 28 days (D28) poststroke (*P* < 0.05; Figure 3(c)). HIIT appeared to be more effective to recover aerobic fitness than MOD as indicated by changes in *S*_max_ and *S*_LT_. Before training, *S*_LT_ and *S*_max_ in the injured groups were significantly lower compared to those in the sham group (*P* < 0.01; Figure 3(d)). After 3 weeks of treadmill training (D28), *S*_LT_ and *S*_max_ increased significantly in the HIIT group (*P* < 0.05; Figure 3(e)). However, no difference was detected between MCAO and MOD groups. In addition, under the same energy expenditure, the total exercise time of the HIIT group was significantly shorter than that of the MOD group, which indicates that HIIT is time efficient and might not be a major obstacle due to its mild exercise tolerance.

### 3.3. Amelioration of Muscle Wasting and Motor Dysfunction after Exercise Training

Furthermore, we assessed the effect of different exercise protocols on skeletal muscle. Measurement of the muscle weight of paretic gastrocnemius mass and the ratio of gastrocnemius mass to body weight indicated that 3 weeks of HIIT protected against muscle loss induced by cerebral ischemia (*P* < 0.01), whereas MOD did not induce body weight significant changes (Figures 2(a), 2(d), and 2(e)). In the functional tests, the results concerning the muscle strength and motor coordination of mice subjected to MCAO after intervention are shown in Figure 2. In the grip strength test, the MCAO group committed a significant decrease (*P* < 0.01) in the right forelimb and a nonsignificant decrease in the left forelimb when compared to the sham (Figures 2(b) and 2(c)), evidencing that the MCAO led to the impairment of the contralateral front paws. Similar to the ladder rung walking test, the MCAO group committed more slip errors in the forelimb (*P* < 0.01) but not in the hindlimb (Figures 2(h) and 2(i)). In the rotarod test and cylinder test, a lower time to fall and higher laterality index were observed in the MCAO group in relation to the other groups (*P* < 0.05; Figures 2(f) and 2(g)), suggesting impairment of the muscle strength and motor coordination due to cerebral ischemia. Based on the performance test conducted before and at the end of the training program, both HIIT and MOD significantly improved the forelimb grip strength and reduced the forelimb slip errors (*P* < 0.05; Figures 2(b), 2(c), and 2(h)). In the rotarod test and cylinder test, HIIT, rather than MOD, significantly improved times to fall and reduced laterality index (*P* < 0.01; Figures 2(f) and 2(g)), indicating that HIIT was superior to MOD with regard to improving the motor function after cerebral ischemia. Both HIIT and MOD can significantly improve the low movement distance and movement speed induced by MCAO (*P* < 0.01; Figure 2(j)).

### 3.4. High-Intensity Interval Training Improved Gait and Pulmonary Functional Parameters

**Figure 4:**
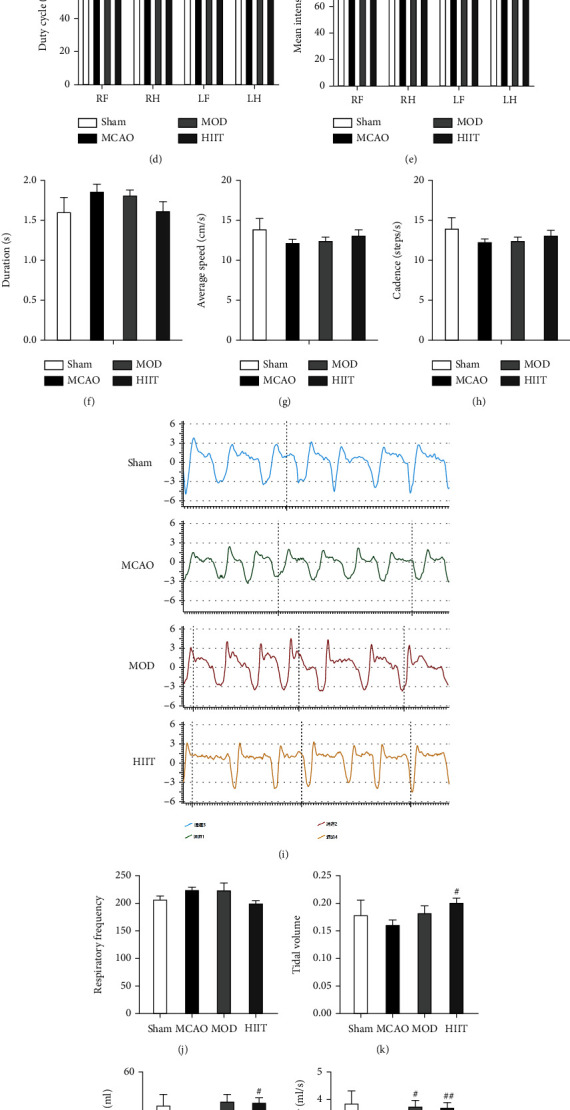
High-intensity interval training improved gait and pulmonary functional parameters. (a) Schematic diagram of labeled footprint and 3D footprint intensity charts. Graph demonstrates statistical differences in the average run characterization parameter in each group, including stand (s) (b), swing (s) (c), duty cycle (%) (d), mean intensity (e), duration (s) (f), average speed (cm/s) (g), and cadence (step/s) (h). (i) The respiration curve illustrates the changes of volume of the plethysmograph induced by the volatility of the mouse's thorax in each group. The graph demonstrates statistical differences in the average pulmonary functional parameter in each group, including respiratory frequency (j), tidal volume (k), minute ventilation (mL) (l), peak inspiratory flow (mL/s) (m), peak expiratory flow (mL/s) (n), and enhanced pause (o). Values are expressed as the mean ± SEM of the mean. ^∗^*P* < 0.05 and ^∗∗^*P* < 0.01 compared with the sham group; ^#^*P* < 0.05 and ^##^*P* < 0.01 compared with the MCAO group as determined by one-way ANOVA (Tukey's multiple comparison test) for the data with normal distribution. The letters for no significance were not shown. Abbreviation: RF: right forelimb; RH: right hindlimb; LF: left forelimb; LH: left hindlimb.

To evaluate the extent of gait impairment after MCAO, we analyzed the run parameters in four groups using the CatWalk automated gait analysis system at 28^th^ day poststroke. The MCAO group displayed obvious but nonsignificant differences from the sham group in period of stand and swing, duty cycle, and mean intensity parameters in the paretic side (Figures 4(b)–4(e)). Furthermore, parameters of duration, average speed, and cadence remained similar between MCAO and sham groups, with minor differences displayed after 3 weeks of HIIT or MOD (Figures 4(f)–4(h)). There was a significant decrease in duty cycle and mean intensity in the right hindlimb after HIIT (*P* < 0.05; Figures 4(d) and 4(e)). From these parameters, it is clear that despite the slight differences observed in walking competency, which might be due to a natural recovery of the animals after cerebral ischemia, HIIT still has the potential to improve certain gait parameters.

The walking energy cost of stroke patients is increased by approximately 1.5- to 2.0-fold that of normal individuals [19]. Therefore, improving cardiorespiratory fitness is an important factor in restoring walking ability. Whole-body barometric plethysmography was used in the present study to noninvasively assess baseline pulmonary function in mice. The results showed that PIF were significantly lower in the MCAO group than those in the sham group (*P* < 0.05; Figure 4(m)). Compared with the MCAO group, PEF and PIF were significantly higher in the HIIT and MOD groups (*P* < 0.05; Figures 4(m) and 4(n)). TV and MV were only significantly higher in the HIIT group (*P* < 0.05; Figures 4(k) and 4(l)), rather than the MOD group.

### 3.5. Effects of Different Exercise Protocols on Paretic Gastrocnemius Muscle Histopathology

**Figure 5:**
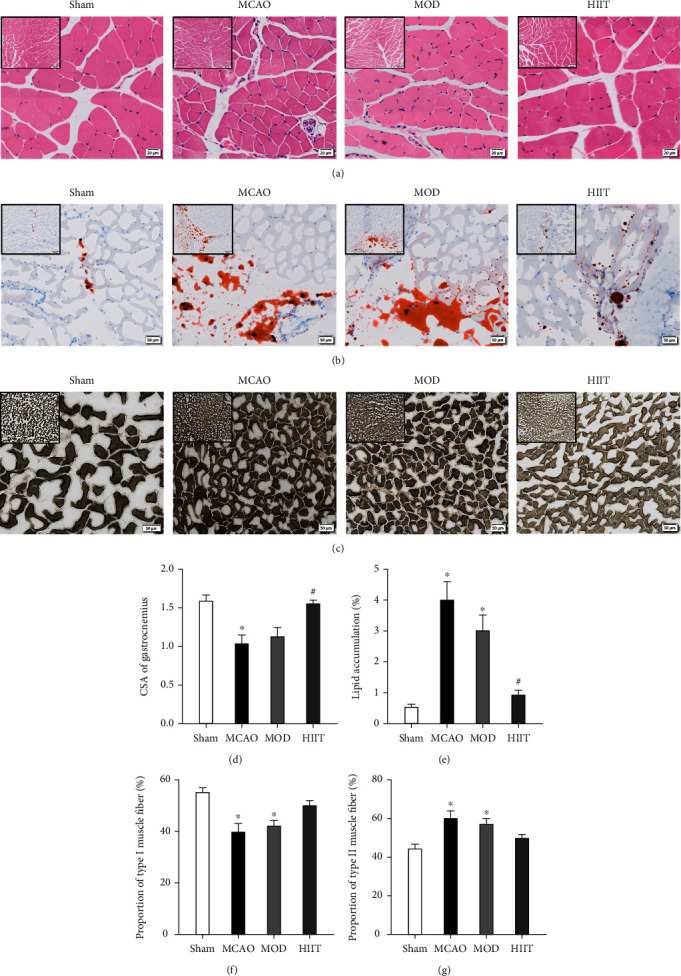
Effects of different exercise protocols on paretic skeletal muscle histopathology. (a) Histological images of HE staining of paretic gastrocnemius muscle tissue in each group (scale bar: 20 *μ*m). (b) Histological images of ORO staining of paretic gastrocnemius muscle tissue in each group (scale bar: 50 *μ*m). (c) Representative gastrocnemius ATP-ase staining in each group (type I fiber: light gray or colorless; type II fiber: dark gray or black) (scale bar: 50 *μ*m). (d) Cross-sectional area (CSA) of paretic gastrocnemius muscle fiber in each group (*n* = 5). (e) Percentage of lipid accumulation in each group (*n* = 5). (f) Proportion of type I muscle fiber in each group. (g) Proportion of type II muscle fiber in each group (*n* = 5). Values are expressed as the mean ± SEM of the mean. ^∗^*P* < 0.05 and ^∗∗^*P* < 0.01 compared with the sham group; ^#^*P* < 0.05 and ^##^*P* < 0.01 compared with the MCAO group as determined by one-way ANOVA (Tukey's multiple comparison test) for the data with normal distribution. The letters for no significance were not shown.

Morphology of the muscle was evaluated by HE staining. The normal muscle fibers observed in the sham-operation group were polygonal, arranged together to form muscle bundles. Compared with the sham group, the MCAO group was characterized by small muscle fiber volume, degeneration, uneven fiber distribution, uneven shape, and a significant decrease in average fiber cross-sectional area (CSA) (*P* < 0.05). In addition, compared with sham-operated mice, fibrosis, inflammatory cell infiltration, increased collagen fiber area, and decreased muscle fiber diameter were detected in the paretic gastrocnemius muscle. Three weeks of HIIT, rather than MOD, significantly alleviated stroke-induced reduction in fiber CSA and the infiltrating inflammatory cells (*P* < 0.05). Collectively, the results indicated that HIIT reduced the stroke-induced muscle atrophy by preventing the reduction in cross-sectional area of the muscle fibers of the paretic gastrocnemius muscle (Figures 5(a) and 5(d)).

ORO staining confirmed the massive accumulation of fatty components in the paretic gastrocnemius of the MCAO group (*P* < 0.05), which was reduced dramatically in the HIIT group (*P* < 0.05), instead of the MOD group (Figures 5(b) and 5(e)).

To ascertain whether different exercise protocols produced distinct impacts on fast and slow muscle fibers, we performed ATP-ase staining for slow and fast myosin heavy chain (MyHC) (Figure 5(c)). The proportion of type II fibers increased significantly (with a consequent reduction in type I fibers) in the paretic gastrocnemius muscle in the MCAO group (*P* < 0.05). No significant difference in the proportion of type I and type II fibers was observed between the MCAO and exercise groups. However, the HIIT group presented a greater trend in promoting muscle phenotypic metastasis compared with the MOD group (Figures 5(c), 5(f), and 5(g)).

### 3.6. Profiling of Differentially Expressed Genes of Skeletal Muscle after Stroke and Exercise Protocols

**Figure 6:**
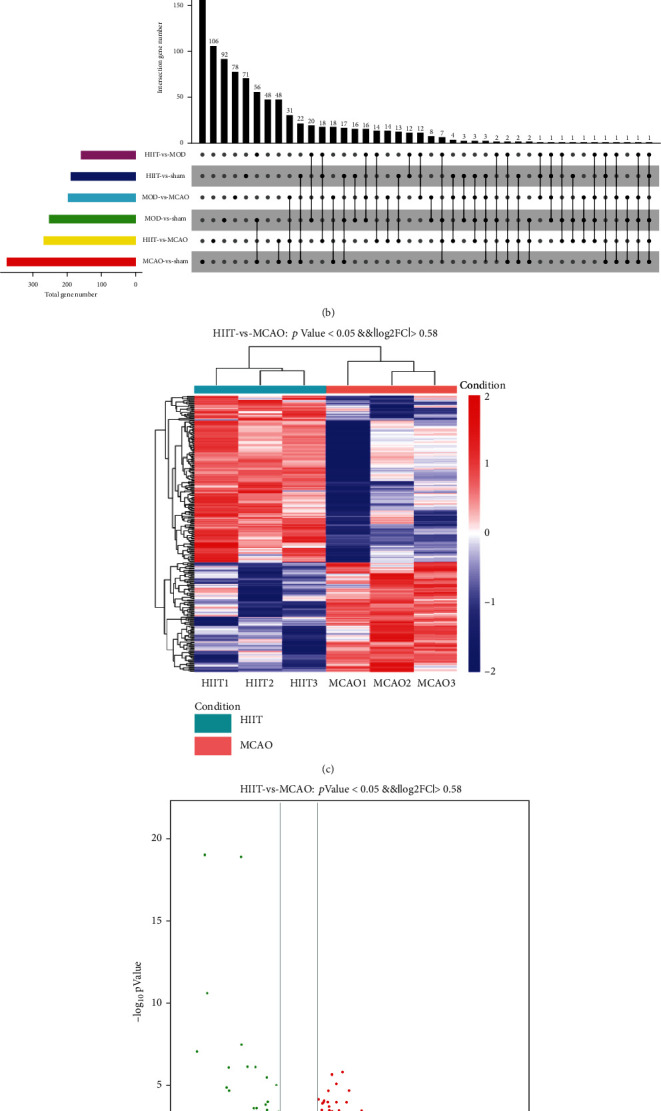
Profiling of differentially expressed genes of skeletal muscle after stroke and exercise protocols. (a) Heat map of differentially expressed genes in each group associated with GO biological process categorization about “atrophy of muscle,” “skeletal muscle fiber type,” and “fatty acid oxidation.” (b) Venn graph illustrates common and unique differentially expressed genes between different comparison groups. (c) Heat map of differentially expressed genes between MCAO and sham groups (dependent group *t*-test *p* < 0.05 and fold difference ≥ 1.5). Red indicates relatively high expression of protein-coding genes, and blue indicates relatively low expression of protein-coding genes. (d) Volcano map of differentially expressed genes between MCAO and sham groups. Gray represents the genes with insignificant difference. Red and green represent the genes with significant difference. (e) KEGG pathway analysis of differentially expressed genes, including cellular processes, environmental information processing, genetic information processing, human diseases, and metabolism organismal systems. Abbreviation: GO: Gene Ontology; KEGG: Kyoto Encyclopedia of Genes and Genomes.

In order to better explore gene expression changes in skeletal muscle after stroke and investigate molecular origin of the pathophysiological process after different exercise regimens, transcriptome-wide RNA sequencing technology was used followed by further bioinformatics analysis. We found 376 differentially expressed genes with 84 and 292 genes up- and downregulated, respectively, in poststroke muscle, and 269 differentially expressed genes with 162 and 107 genes up- and downregulated, respectively, between HIIT and MCAO groups (Figure 6(b)). These results are visualized via volcano plot and heat map depicting differentially expressed genes (Figures 6(c) and 6(d)). To obtain more detailed gene expression patterns, selected differentially expressed genes associated with GO biological process categorization were shown in the heat map, including “atrophy of muscle,” “skeletal muscle fiber type,” and “fatty acid oxidation” (Figure 6(a)).

**Figure 7:**
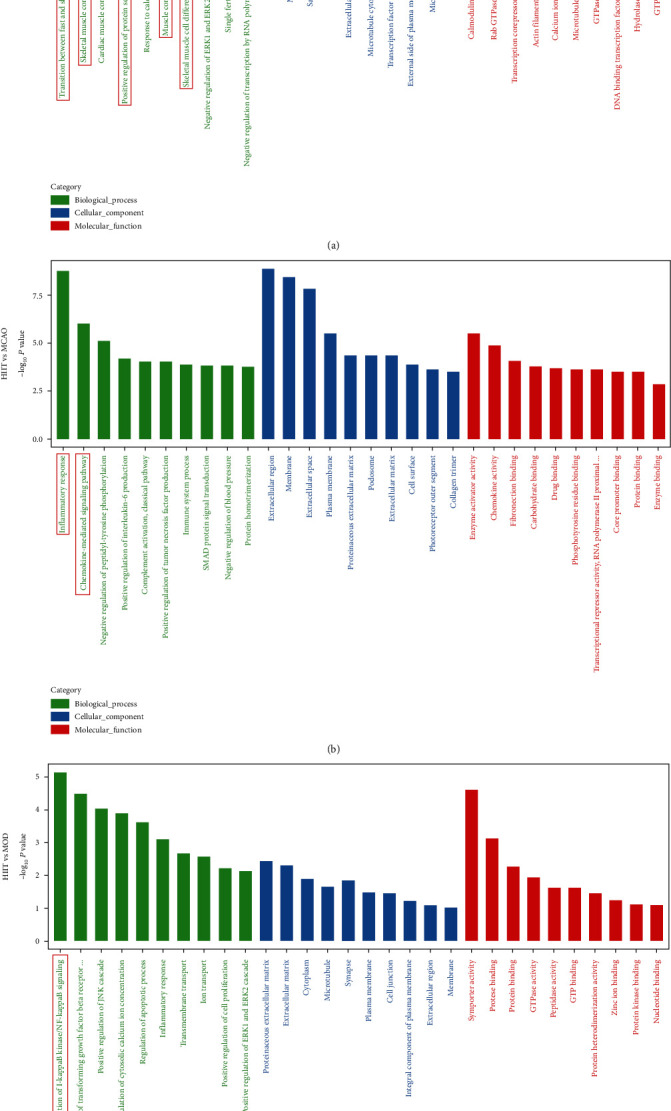
GO term enrichment analysis of differentially expressed genes, including cellular component, biological process, and molecular function. (a) GO annotation analysis between MCAO and sham groups. (b) GO annotation analysis between HIIT and MCAO groups. (c) GO annotation analysis between HIIT and MOD groups. Abbreviation: GO: Gene Ontology.

We used GO annotation analysis and KEGG enrichment analysis of differentially expressed genes to characterize their respective biological functions. Our GO annotation analysis was assigned terms in the biological process, cellular component, and molecular function, respectively. Most biological-process-related genes between MCAO and sham groups were annotated with GO terms associated with “transition between fast and slow fiber,” “skeletal muscle contraction,” “positive regulation of protein secretion,” and “skeletal muscle cell differentiation” (Figure 7(a)). The most significantly enriched pathways by KEGG enrichment analysis were “immune system,” “lipid metabolism,” “infectious diseases,” “signal transduction,” and “transport and catabolism” (Figure 6(e)), indicating that the differentially expressed genes of skeletal muscle after stroke were highly associated with signaling pathways involved in inflammatory response.

Furthermore, most biological-process-related genes between HIIT and MCAO groups were annotated with GO terms associated with “inflammatory response” and “chemokine-mediated signaling pathway” (Figure 7(b)). Most biological-process-related genes between HIIT and MOD groups were annotated with GO terms associated with “regulation of I-kappaB kinase/NF-kappaB signaling” (Figure 7(c)). The results of our GO and KEGG enrichment analysis indicated that HIIT modulated inflammatory response activation and atrophy of muscle induced by stroke might be via the NF*κ*B involved signaling pathway and exerting an anti-inflammatory effect in skeletal muscle.

### 3.7. Level of Cytokines in Paretic Skeletal Muscle and Lymphocyte Subsets in Peripheral Blood

**Figure 8:**
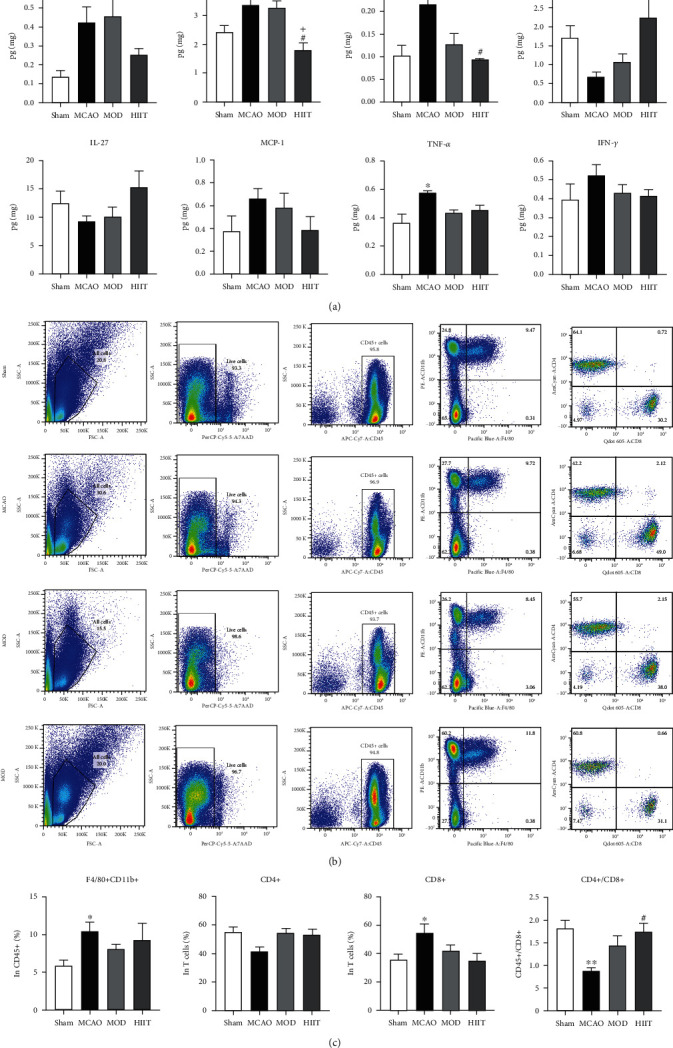
Level of cytokines in paretic skeletal muscle and lymphocyte subsets in peripheral blood. (a) The profiles of multiple cytokines in paretic gastrocnemius muscle in each group, including interleukin- (IL-) 1*α*, IL-1*β*, IL-6, IL-10, IL-27, monocyte chemoattractant protein (MCP-1), tumor necrosis factor- (TNF-) *α*, and interferon- (IFN-) *γ*. (b) Representative fluorescence activated cell sorting (FACS) plots of F4/80+CD11b+ macrophages gated on CD45+ cells, CD4+ cells gated on CD45+CD3+ cells, and CD8+ cells gated on CD45+CD3+cells in muscle tissue. (c) Bar graph showing the percentage of F4/80+CD11b+ cells in CD45+ cells, CD4+ cells in CD45+CD3+ cells, CD8+ cells in CD45+CD3+ cells, and the ratio of CD4+ and CD8+ cells. Values are expressed as the mean ± SEM of the mean. ^∗^*P* < 0.05 and ^∗∗^*P* < 0.01 compared with the sham group; ^#^*P* < 0.05 and ^##^*P* < 0.01 compared with the MCAO group; ^+^*P* < 0.05 and ^++^*P* < 0.01 compared with the MOD group as determined by one-way ANOVA (Tukey's multiple comparison test) for the data with normal distribution. The letters for no significance were not shown.

Thus, we profiled the levels of 13 cytokines, using the bead-based immunoassay LEGENDplex, to explore the local inflammatory status in paretic gastrocnemius muscle. The proinflammatory cytokines are involved in the process of muscle loss, including IL-6 and TNF-*α*, which were significantly elevated in the MCAO group compared with sham-operated mice (*P* < 0.05). HIIT significantly reduced the levels of IL-1*β*, IL-6, and elevated IL-10 level compared with the MCAO group (*P* < 0.05). There were no significant differences found in the above cytokines between MOD and MCAO groups (Figure 8(a)).

Flow cytometry analysis results showed that mice subjected to MCAO induced a significant increase in CD8+ T cells and F4/80+CD11b+ cells, the well-known surface marker of mouse macrophages (*P* < 0.05). Although both CD4+ T cell and CD8+ T cell proportions were not significantly altered, HIIT remarkably elevated the ratio of CD4+ and CD8+ T cells (*P* < 0.05). Unlike the HIIT group, MOD failed to significantly affect the proportion of macrophages and T lymphocyte subsets in paretic gastrocnemius (Figures 8(b) and 8(c)).

### 3.8. HIIT Modulated Macrophage-Mediated Inflammation and Cytotoxic Properties via Inhibiting the TLR4/MyD88/NF*κ*B Signaling Pathway

**Figure 9:**
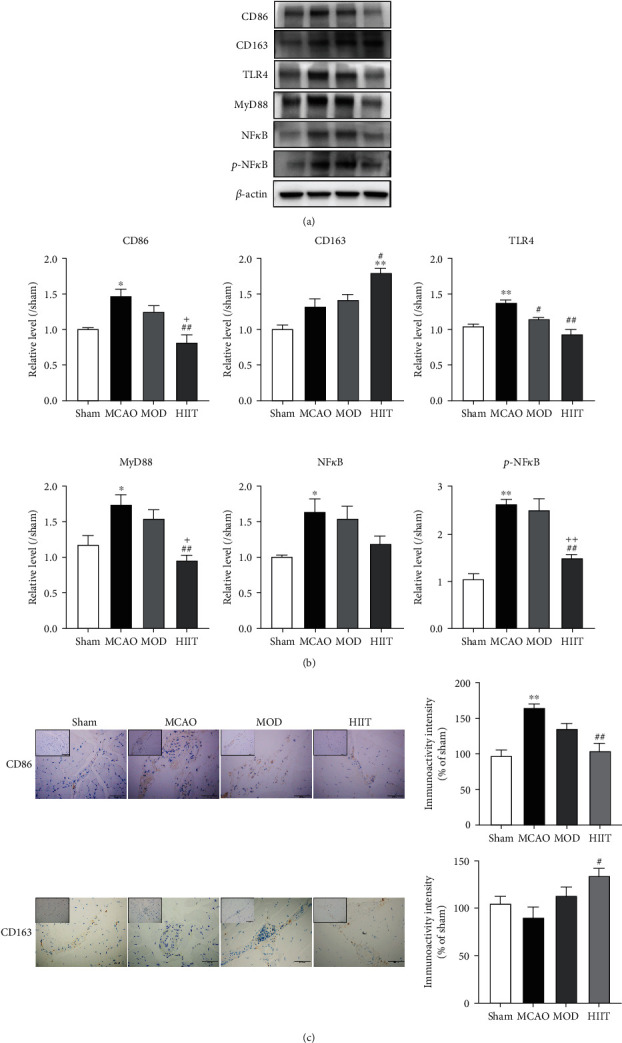
HIIT modulated macrophage-mediated inflammation and cytotoxic properties via inhibiting the TLR4/MyD88/NF*κ*B signaling pathway. (a, b) Representative immunoblots and quantification normalized to sham group condition of CD86, CD163, TLR4, MyD88, NF*κ*B, p-NF*κ*B, and *β*-actin protein level (*n* = 5). (c) Representative immunostaining and quantification of CD86 and CD163 in paretic gastrocnemius tissues (*n* = 5). Values are expressed as the mean ± SEM of the mean. ^∗^*P* < 0.05 and ^∗∗^*P* < 0.01 compared with the sham group; ^#^*P* < 0.05 and ^##^*P* < 0.01 compared with the MCAO group; ^+^*P* < 0.05 and ^++^*P* < 0.01 compared with the MOD group as determined by one-way ANOVA (Tukey's multiple comparison test) for the data with normal distribution. The letters for no significance were not shown.

Since training protected against inflammatory response induced by stroke directly, we examined the effect of HIIT on the infiltrating immune cells in paretic skeletal muscle. Macrophages mediate the recruitment and activation of systemic immune cells and induce cytotoxicity in chronic inflammation. We, therefore, examined the macrophage profile in skeletal muscle poststoke. The gene expression of subsets of macrophages (M1/CD86 and M2/CD163, respectively) is demonstrated in relative expression of *β*-actin (Figure 9). Western blotting and immunostaining showed that MCAO induced a significant increase in the number of cytotoxic CD86+ (M1 type) macrophages compared to the sham group (*P* < 0.05). No differences were noted in the number of CD163+ (M2 type) macrophages between MCAO and sham groups. We further confirmed that HIIT, instead of the MOD group, significantly decreased the number of CD86+ macrophages and increased the number of CD163+ macrophages in the paretic gastrocnemius muscle compared to the MCAO group (*P* < 0.05; Figure 9).

To further delineate the mechanisms potentially involved in the modulating of macrophage phenotypes after HIIT, we hypothesized that the TLR4/MyD88/NF*κ*B signaling pathway might be a crucial regulator of skeletal muscle atrophy, as evidenced by the result of GO and KEGG enrichment analysis above. Expression of TLR4, MyD88, NF*κ*B, and p-NF*κ*B was all significantly increased in the paretic gastrocnemius muscle of the MCAO group (*P* < 0.05). Notably, HIIT downregulated the expression of TLR4/MyD88/NF*κ*B signal to control levels (*P* < 0.05; Figures 9(a) and 9(b)), indicating normal reactivity, whereas there were no differences between MOD and MCAO groups.

## 4. Discussion

This study investigated the effects of high-intensity interval and moderate-intensity aerobic training on muscle mass, strength, physical function (walking competency and cardiorespiratory fitness), and stroke-related gene expression profile of skeletal muscle in a preclinical mouse model of cerebral ischemia. We originally demonstrated that both low volume HIIT and MOD prevented stroke-related skeletal muscle mass loss in mice. HIIT was more beneficial than MOD for improvements in walking competency and cardiorespiratory fitness. Additionally, RNA-seq analysis revealed that the differentially expressed genes between HIIT and sedentary MCAO groups were highly associated with signaling pathways involved in inflammatory response. Following the outcome, we further investigated the infiltrating immune cells abundant in paretic muscles. The results showed that HIIT modulated macrophage activation by stimulating M1-to-M2 polarization via inhibiting the TLR4/MyD88/NF*κ*B signaling pathway, thus exerting an anti-inflammatory effect in paretic skeletal muscle. It is expected that these data will provide novel insights into the mechanisms and potential targets underlying muscle wasting in stroke.

The MCAO model is characterized by high reproducibility and large infarct volumes involving a substantial proportion of the hemisphere (including most of the cortex, striatum, thalamus, hippocampus, and subventricular zone) [20]. Muscle loss or weakness is associated with these motor system impairments due to the nerve fiber degeneration of the motor cortex, striatum, internal capsule, and the descending projection pathways, as well as reduced muscle activation and incoordination [21]. Loss of muscle in the nonparetic limbs is also probable over time as stroke survivors are known to have a sedentary lifestyle [11]. It has been reported that patients with acute stroke were physically active for less than 40 min a day during hospitalization [22]. The open field test in this study also showed that the distance moved and mean velocity of MCAO mice significantly decreased compared with the sham group and the other two exercise groups.

Inactivity and immobilization after stroke are important factors of muscle loss or decreased fiber cross-sectional area as muscle unloading produces a multitude of maladaptive responses, such as insulin resistance, glucose-dependent energy metabolism, and intramuscular lipid disposition [23]. A recent systematic review based on 7 studies involving 1695 stroke patients showed that the prevalence of stroke-related sarcopenia within 1 month was 50% and that at 6 months was 34% [8]. In the present study, the paretic gastrocnemius mass was lower in the MCAO group than that in the sham group until the 4^th^ week postischemia. Next, we calculated the ratio of gastrocnemius mass to body weight. The MCAO group also showed a significant decrease in the ratio relative to the sham group. These results corroborate the previous study predicting that adaptive responses in muscle tissue will be most pronounced in the early phase after stroke [23]. Skeletal muscles make up 40% of the body's mass. It is taken for granted that the loss of muscle mass after stroke is accompanied by weight reduction. A significant decrease in the body weight of animals with cerebral ischemia was observed in the present study as early as 4 days after stroke, which is consistent with those reported by Modo et al. [24], where ischemic animals with cerebral ischemia presented a lower weight gain few days after surgery relative to the controls. Compared with the nonexercise group, both 3 weeks of HIIT and MOD significantly reversed the ratio of gastrocnemius mass to body weight. However, HIIT but not MOD significantly improved the weight gain rate at 2^nd^ week after cerebral ischemia and finally contributed significantly to restore paretic gastrocnemius mass after 3 weeks of training.

Recently, there have been suggestions that low muscle strength, rather than low muscle mass, be considered the major determinant of sarcopenia [5]. It is worth noting that the loss of muscle tissue may be replaced by intramuscular lipid configuration, so the actual reduction of functional muscle tissue may be higher than inferred from simple weight assessment. A meta-analysis included 11 trials that report slightly greater fat mass in the paretic arms compared to nonparetic arms in stroke survivors. Although there is no significant increase in whole-body fat mass from 1 to 6 months poststroke, it does increase between 6 and 12 months after the stroke [25]. Herein, our data also indicated that massive lipid droplet accumulation in paretic gastrocnemius was detected by Oil-Red-O staining in the MCAO sedentary group, and it was HIIT but not MOD that significantly reduced the lipid disposition. Skeletal muscle energy flux during contraction is intensity dependent. At the same time, high-intensity exercise was the most efficient exercise regimen regarding depleting glycogen stores, elevating fat oxidation rates, and promoting lipid mobilization and energy expenditure in the postexercise period compared to low- to moderate-intensity exercise [26, 27].

Indeed, skeletal muscle mass deficit more appears to be an independent predictor of poor outcome after stroke. Ohyama et al. [28] revealed that the presence of the skeletal muscle mass defects in over half of patients with acute ischemic stroke, who tended to display worse conditions (i.e., severe neurological impairments and poorer functional outcome) at admission and longer hospital stay. A growing body of evidence supports regular physical exercise as the most effective strategy for improving sarcopenia and physical function [11]. Nevertheless, it is unclear whether the positive effects of exercise interventions can be sustained for an adequate period and maintained at sufficient intensity to prevent incident disabilities [29]. In the present study, a remarkable decrease of forelimb grip strength and latency to fall, as well as a significant increase of laterality index and forelimb slip error percent, was observed in ischemic animals. Similar to muscle mass, both HIIT and MOD reversed the decline in muscle strength of hemiplegic limbs and improved the physical function. In fact, for most people, greater health benefits can be obtained by engaging in intensive or longer physical exercise [30, 31].

Walking performances are important for stroke patients to maintain independent living and participate in family, social activities [32]. Gait abnormalities along with muscle weakness place stroke survivors at a high risk of falls. A recent meta-analysis showed that compared with low-to-moderate-intensity exercise or regular physical activity, high-intensity exercise may be a safe and more effective stimulus to improve the walking ability of stroke patients, with improved walking distance, comfortable gait speed, and stride length [32]. Gait analysis in the present study showed that HIIT can effectively reduce the duty cycle and mean intensity of the right hindlimb. Many stroke patients may be restricted in their daily activities because of their adverse events related to cardiorespiratory fitness, which is considerably low poststroke, with VO_2_ peak values ranging from 8 to 22 mL/kg/min, equivalent to 26%~87%, respectively, of that of healthy age- and sex-matched healthy individuals [33]. Cardiorespiratory fitness reflects the ability of circulatory and respiratory systems to supply oxygen for skeletal muscles during moderate- to high-intensity exercise training [34]. Our study reported that HIIT induced a significant increase in tidal volume, minute ventilation, peak inspiratory flow, and peak expiratory flow, while MOD failed.

Human skeletal muscle fibers have great adaptive potential; however, the molecular mechanism of atrophy and phenotypic transition after stroke is not clear [35]. Slow myosin heavy chain (MHC) type I fibers are characterized by a large number of mitochondria, oxidative metabolism, and fatigue resistance. Conversely, fast oxidation glycolysis IIA fiber and glycolysis IIb/X fiber have lower fatigue resistance, and their energy mainly derives from anaerobic glycolysis [36]. In normal aging, muscle fibers shift from fast to slow fibers with more reliance on anaerobic metabolism, resulting in a reduction of muscle strength [37]. In contrast, different from age-related sarcopenia, the characteristic of stroke-related muscle alterations is a slow-to-fast muscle fiber shift, which was a strong predictor of impaired function, such as gait disorders poststroke [7]. In elderly stroke patients, fast type IIx and IIa MHC fibers in paretic vastus lateralis significantly increased compared with nonparetic muscle. The proportion of these fibers is only negatively correlated with the self-selected gait speed of the paralyzed leg [38]. In the present experiment, an exercise protocol of high intensity was applied for continuous 3 weeks, which caused a considerable but nonsignificant increase in the proportion of type I fibers and a decrease in the proportion of type II fibers. Yan et al. [39] reported that sports training may lead to an increase in the proportion of MHC I type fibers in skeletal muscle, but only in the cases of long-term exercise, such as athletes who undergo intense training for years.

In this study, we further used mRNA-seq to determine the effect of different exercise programs on skeletal muscle gene expression of the MCAO mice. Gene ontology analysis implied that signaling pathways involved in inflammatory response might contribute to the protein synthesis and degradation of muscle. According to a different and emerging research and consistent with our results, stroke-related sarcopenia may be associated and even caused by inflammation [40, 41]. A large body of literature showed that inflammatory cytokines activate many molecular pathways involved in skeletal muscle consumption, resulting in the imbalance between protein synthesis and catabolism [42, 43]. Our findings may suggest that the plasma titer of some inflammatory molecules (IL-1*β*, IL-6, TNF-*α*, and IL-10), which are important cytokines related to the regulation of Th1 and Th2 implicated in skeletal muscle regeneration through myogenic and myeloid cell activation, could be related to muscle decline and functional impairment [27]. Acute exercise can increase the plasma level of the same proinflammatory cytokines, possibly due to stress response, whereas regular exercise seems to upregulate the anti-inflammatory ability, leading to a decrease in the level of systemic inflammation and circulating inflammatory markers [44, 45]. Extensive studies have driven the discussion about the anti-inflammatory effect of exercise that may be mediated (to some extent) by some activities of myokines released into the blood during long-term contraction [44]. The production and subsequent release of myokines into the circulation seem to be directly related to the duration and intensity of training. This thesis is supported by our results that HIIT determines the decrease of IL-6 and IL-1*β* levels, which do not reach a continuous level of diminution within low to moderate intensity of training [46].

Among infiltrating immune cells in muscle, macrophages play a central role in the activation and protection of muscle fibers after muscle inflammation and injury [47]. In addition, some studies have found that there are a large number of resident macrophages in the adventitia and fascicular membrane, which control the immune responses in the process of muscle injury [48, 49]. Although the phenotypes of macrophages are heterogeneous in various tissue and environments, there are two phenotypes of activated macrophages, namely, proinflammatory M1 and anti-inflammatory M2. M1 (classical activation) macrophages mainly secrete inflammatory cytokines, including IL-1*β*, IL-6, TNF-*α*, ROS, NO, and MMP, which in turn promote CD4+/Th1 cells that enter muscle from the blood circulation, consequently causing acceleration of myofiber lysis and protein degradation. The M2 macrophages (alternative activation) abundant during the late phase of tissue repair can release anti-inflammatory cytokines, such as TGF-*β*, IL-10, and IGF-1, thus prompting the anti-inflammatory effect of CD8+/Th1 cells, which are mainly involved in the phagocytosis and cleaning of the injured site, contributing to myogenesis and tissue repair [50, 51]. In this study, CD86 and CD163 were detected as markers of M1 macrophages and M2 macrophages, respectively. We observed the increase of M1 macrophages and the decrease of M2 macrophages in the paretic gastrocnemius muscle of ischemic mice. Therefore, a definitive understanding of the complex temporally coordinated macrophage roles in stroke-related sarcopenia and the balance of M1 and M2 macrophages is crucial in muscle recovery [10].

Emerging evidence shows that moderate training regulates macrophage activation by stimulating M1 to M2 polarization and playing a global anti-inflammatory role in multiple organs [52, 53]. In skeletal muscle, physical activity stimulates the release of myokines related to M1/M2 ratio regulation (e.g., IL-6, TNF-*α*, and IL-10), which is involved in skeletal muscle regeneration [54, 55]. The effect of exercise strongly depends on its modality, intensity, and timing. A recent study investigating the direct effects of high-intensity continuous training (HICT) on neuroprotection in the central nervous system (CNS) suggested that HICT protected the CNS against autoimmune neuroinflammation by reducing microglial-derived neurotoxicity, and proinflammatory responses, rather than inducing their shift to M2 phenotype [56]. However, it has also been observed that excessive vigorous exercise still promoted M2 polarization of macrophages in skeletal muscle, and myogenesis increases despite the increase of TNF-*α* [57]. In our study, results indicated that after 3 weeks of HIIT, rather than MOD, inhibition of M1 phenotype occurred concomitantly with the increase of M2 macrophage marker, and further pathway analyses implicated changes in the regulation of I*κ*B kinase/NF*κ*B signaling between HIIT and MOD groups, which may contribute to modulating M1-to-M2 polarization.

Ample evidence shows that macrophage polarization is mainly regulated by the toll-like receptor (TLR) pathway, which plays a critical role in the nonspecific immune response [58]. TLR4, the earliest receptor protein discovered, binds to corresponding ligands and induces cell activation through myeloid differentiation factor 88- (MyD88-) dependent and MyD88-independent pathways, activating the p65 subunit of downstream NF*κ*B to secrete inflammatory factors [53]. Our subsequent results also confirmed that HIIT could downregulate the expression of TLR4, MyD88, and phosphorylation of NF*κ*B, thereby inhibiting the M1 type polarization of macrophage and indirectly promoting the M2 type polarization, which is conducive to restore the dynamic balance between the polarization of M1 and M2. That may be a vital mechanism for HIIT to regulate macrophage polarization in both directions, suppress chronic low-grade inflammation of muscles after cerebral ischemia, and promote muscle repair.

In spite of these findings, there are some limitations to be solved in the future. Firstly, the inflammatory response occurs early soon after stroke, which will impede stroke recovery [59]. Moreover, the endogenous recovery in the chronic phase is usually insufficient to significantly improve long-term functional outcomes. The present study failed to explore the role of inflammation markers and cells in predicting stroke outcome in the early stage. In addition, we did not reveal the long-term effects of different exercise regimens on muscle wasting. Future experiments should be dedicated to solve the above problems in clinic setting. Secondly, the global changes of TLR4/MyD88/NF*κ*B signaling are significant in whole muscle tissue. However, it is better to reveal the alteration of TLR4/MyD88/NF*κ*B signaling by isolating infiltrating macrophages using flow sorting.

## 5. Conclusion

In conclusion, HIIT induces direct beneficial effects on muscle repair and physical function in an experimental model of MCAO compared to MOD. The long-term inflammatory response of muscle after cerebral ischemia serves as a key therapeutic target, and HIIT may regulate macrophage polarization via inhibiting the TLR4/MyD88/NF*κ*B signaling pathway, thereby reducing the cytotoxicity and proinflammatory properties of macrophages. Elucidating the mechanisms underlying the positive effects of exercise training on stroke-related sarcopenia will facilitate the translation of basic research findings to clinical benefits for sedentary patients. Importantly, our study demonstrated the different effects on muscle mass, strength, and gene expression between different exercise regimens in terms of type, intensity, frequency, and duration. Therefore, long-term exercise training and specific exercise program are required to optimally respond to deleterious inflammatory response.

## Data Availability

The datasets used and/or analyzed during the present study are available from the corresponding author on reasonable request.

## Supplementary Materials

Supplementary Materials Supplementary Methods: the concise description of methods and descriptive statistics (Supplemental Table 1, Supplemental Table 2), including behavioral tests, HE staining, immunohistochemistry, RNA sequencing, and differentially expressed gene analysis, profiling of cytokines/chemokines, western blot assays.
