# Supplementary material for: High-Intensity Interval Training Improves Physical Function, Prevents Muscle Loss, and Modulates Macrophage-Mediated Inflammation in Skeletal Muscle of Cerebral Ischemic Mice
Source: Mediators Inflamm. 2021 Nov 20;2021:1849428. doi: 10.1155/2021/1849428 (PMC8627337; doi:10.1155/2021/1849428)
Supplement: Supplementary Materials — Supplementary Methods: the concise description of methods and descriptive statistics (Supplemental Table 1, Supplemental Table 2), including behavioral tests, HE staining, immunohistochemistry, RNA sequencing, and differentially expressed gene analysis, profiling of cytokines/chemokines, western blot assays. [file 1849428.f1.docx]

**Supplementary Methods:**

**High-intensity interval training improves physical function, prevents muscle loss and modulates** **macrophage-mediated inflammation in skeletal muscle of cerebral ischemic mice**

1. **Animals**

Male C57BL/6 mice (20-25g), purchased from Shanghai Jihui Laboratory Animal Care Co., Ltd., were initially housed in standard plastic cages (cage size: 26 × 19 × 15cm) in a temperature-controlled environment (22 ± 2°C) with 50 ± 10% humidity under a 12/12 h light/dark cycle (lights on 7:00 a.m.) with free access to sufficient food and water. All experiment procedures were performed according to the National Institutes of Health guide for the Care and Use of Laboratory Animals. Before use, the mice were allowed to acclimate to laboratory conditions for seven days.

1. **Middle cerebral artery occlusion surgery**

Middle Cerebral Artery Occlusion (MCAO) model of left middle cerebral artery ischemia for 60 minutes was established after anesthesia by 1% pentobarbital sodium (10mg/kg. ip). Incisions were made at the neck midline to expose the left common carotid artery (CCA), external carotid artery (ECA) and internal carotid artery (ICA). After ECA was ligated, a blunt nylon thread (Guangzhou Jialing Co., Ltd, China) was inserted from the left ECA, through the bifurcation of CCA, into the intracranial segment of ICA (10±1 mm away from the carotid bifurcation) to block blood flow at the middle cerebral artery (MCA) for 60min. Then, the nylon thread was withdrawn to allow reperfusion. Finally, the skin was sutured. In the Sham group, CCA, ECA and ICA were just exposed without being inserted by the thread to block the blood flow at MCA[1]. The blood flow of middle cerebral artery was monitored by laser speckle blood flow meter. TTC staining was used to identify the area of infarction. The neurological deficit level of the mice was examined using the modified neurological severity score (mNSS) 24 hours after MCAO surgery. The full score of mNSS is 14 points, including motor (muscle state and abnormal movement), sensory (vision, touch and proprioception), reflex and balance tests. Failure to perform one of the tests will get 1 point, and no corresponding test reflection will deduct 1 point. The overall comprehensive score is used to determine the degree of injury. MCAO mice with mNSS more than 6 participated in the experiment.

After MCAO, mice were excluded according to the following criteria: 1) mNSS less than 6 or overactive (n=17); 2) resisting running on treadmill (n=5); 3) death during or after the surgery and ineffective arterial occlusion (n=12). Overall, 80 mice were used, and 46 of them were included. Mice were randomly assigned to Sham group (n=10), MCAO group (n=12), MOD group (n=12), HIIT group (n=12).

1. **Incremental test and exercise protocols**

The protocol was performed with the following adaptations according to the previous published work[2]. Seven days after MCAO, the mice in HIIT and MOD groups were placed on the motor driven treadmill (Huaibei Zhenghua Biological Instrument Equipment Co., Ltd, China) and warmed up at a rate of 6m/min for 5min. Then accelerate by 3 m/min every 3 minutes until the mouse cannot maintain the applied speed, and the final speed is defined as the maximum speed (Smax). Every 20 seconds of acceleration, a small amount of blood (0.2 μL) was collected from the tail vein. The blood lactate concentration (mmol/L) was measured by a portable blood lactate device (lactate Scout+, EKF diagnostics, Germany). When the blood lactic acid concentration measured twice in a row has a significant inflection point or increased by 1 mmol/L, the blood lactic acid concentration measured in the previous measurement is regarded as the lactic acid threshold (LT), and the corresponding treadmill speed is called S_LT_.

HIIT: The session consists of a 4 x 4-minute high-intensity treadmill run (S_LT_ + 60-70% (Smax-S_LT_)), interrupting active recovery (S_LT_) for 3 minutes between each intensity series. The plan is implemented five times a week for three weeks.

MOD: The speed is fixed at 80-90% S_LT_ to avoid the accumulation of lactic acid in the body. The project is carried out every day for 3 consecutive weeks. In order to match the total energy expenditure (W) between the groups and only compare the effects of intensity, according to the energy expenditure (exercise + recovery) of the HIIT group, the daily exercise time of the MOD group was adjusted by the following formula:

W (J/kg·m) = mass (kg) x speed (m/min) x time (min) x treadmill tilt (°) x 9.8

Each program included a 5 minutes warm-up (50% S_LT_) before the formal training. The Smax and S_LT_ of the rats were re-tested every two weeks to adjust the training intensity. The mice in the sham sedentary groups were housed in a crowded cage (cage size: 26 × 19 × 15cm, 8/per cage) with no access to the treadmill.

1. **Behavioral tests**

The behavioral tests were performed 2 days before and 3 weeks after exercise intervention. The test sequence was as follows:1) Open field test; 2) Grip strength test; 3) Rotarod test; 4) Cylinder test; 5) Ladder rung walking test; 6) CatWalk XT gait test.

**4.1 Open field test**: The experimental device consists of a plastic test box (40 × 40 × 50cm) and an automatic video system. The mouse was placed into the center of the open field to record its free movement for 10 minutes. The distance moved and mean velocity were recorded and analyzed. The box was thoroughly cleaned with 75% alcohol to remove any olfactory cues.

**4.2 Grip strength test**: The grip forces exerted by both forelimbs and by each forelimb separately were quantified by using a grip force tester (YLS-13A, Jinan Yiyan Co., Ltd, China). The mouse was placed on the grip plate gently, and the tail of the mouse was pulled back gently (not jerk). After the mouse grasps the grip plate, pull it back evenly, causing the mouse to loosen its paws. At this time, the instrument will automatically record the mouse’s maximum holding power, accompanied by an audio prompt. The time interval between each trial was fixed to 1 min to avoid fatigue accumulation.

**4.3 Rotarod test**: The pre-training test (in order to establish a performance baseline) is performed 4 times a day for three consecutive days. The rotating rod accelerates from 5 revolutions per minute (RPM) until it rotates at a constant speed after 40 RPM within 90s. Each test lasts for a maximum of 5 minutes, and rests for 3 minutes between tests to avoid fatigue. Record the latency of each mouse falling from the rod in each test, and calculate the average latency as the final result. If the mouse hugs the revolver for 2 laps, stop timing. After training or testing, clean the instrument with alcohol and dry it[3].

**4.4 Cylinder test**: Mouse was placed in a transparent cylinder (10 cm in diameter and 15 cm in height), and record the use of forelimbs in the exploratory behavior within 5 minutes. Place a mirror at the appropriate position of the cylinder to ensure that the forelimb activity can be recorded even when the mouse was turned back to the camera lens. The evaluator uses a video recorder with slow-motion and clear freeze-frame functions to record and score. When analyzing the behavior in the cylinder, record the number of times that the right paw, the left paw, and both paws touch the cylinder wall simultaneously. Data were analyzed using asymmetry, which was calculated as follows: (left + 0.5×both) / (right + left + both) ×100%[3].

**4.5 Ladder rung walking test**: Mouse was trained to pass through a ladder device (70 cm in length and 15 cm in height) inserted with multiple iron rods (0.2 cm in diameter) arranged in parallel. The first 30cm, the spacing of each iron bar is 1cm, and the last 40cm, the spacing of each iron bar is 2cm. The whole device is placed on two supports 17 cm high from the ground. Before MCAO, mouse was trained to cross ladders for three consecutive trials. The number of times that the forelimb and hindlimb slipped from the iron bar when the mouse crossed the ladder was recorded continuously with a camera[3].

**4.6 CatWalk XT gait test**: In this study, a 150cm-long small animal glass track CatWalk XT (Noldus, Netherlands, Wageningen) system was used. A high-speed camera was placed under the glass plate to capture images of mouse paw changes for gait data collection. In the experiment, the mouse was placed on the runway and passed smoothly, and it had to be completed within 8 seconds. The maximum speed change was 60%. At least 3 movement averages that met this standard were recorded in each round. The gait analysis indicators selected in the study included standing time, limb swing time, duty cycle, average running speed, stride length, mean intensity of the complete paw. In addition, the average running speed and running time of mice were analyzed as a whole[4].

1. **Pulmonary function**

The pulmonary function of mice was evaluated by whole-body plethysmograph. Briefly, three days before the test, mice were placed in a sealed box that was connected to transducers and a computer and allowed to acclimate for 15 min every day. On the test day, the mice were placed again in the box to acclimate for 5min. Afterwards, pulmonary function was recorded and assessed for 5min, including peak expiratory flow (PEF), peak inspiratory flow (PIF), and minute volume (MV), tidal volume (TV).

1. **Body weight and muscle mass**

The body weight of the mice was measured at fixed times every 4 days until the end of the experiment, and the body weight growth rate was calculated as: growth rate (%) = (current body weight - body weight at the first day) / body weight at the first day x 100%. Paretic gastrocnemius muscle of mice in four groups were isolated, removed and weighed after deeply anesthetized.

1. **Experimental material preparation**

Mice were deeply anesthetized by 1% pentobarbital sodium (10mg/kg. ip). For western blotting, RNA Sequencing, adenosine triphosphate (ATP)-ase staining and Oil-Red-O staining (n = 7 each group), mice in each group were sacrificed and paretic gastrocnemius were quickly removed, placed in Eppendorf tubes, frozen in liquid nitrogen, and stored at -80°C for further use. For immunohistochemistry, the mice (n=5 each group) were transcardially perfused with 50mL of phosphate buffered saline (PBS) and then fixed with 50mL 4% paraformaldehyde (PFA) solution. The paretic gastrocnemius was removed, post-fixed for 24h in the same fixative and cryoprotected 24h at 4˚C in 30% sucrose solution. After then, the tissue blocks were embedded in paraffin for further use. Blood sample (300 μL) the blood was collected by enucleating the mouse eyeball for flow cytometric analysis.

1. **HE staining and immunohistochemistry**

HE staining paraffin sections xylene dewaxing, gradient ethanol hydration, hematoxylin-eosin stain, then gradient alcohol and xylene dehydration, mount. For immunohistochemistry, the serial coronal sections were then affixed onto the amino-propyl-tri-ethoxy-silane (APES) coated slides and incubated at 60°C for 2 hours after drying naturally. And then the slides were incubated with 3% H_2_O_2_ at room temperature for 10min after deparaffinized and rehydrated. The sections were treated 10min with sodium citrate buffer (10mM Sodium Citrate, 0.05% Tween 20, pH 6.0) in the pressure cooker for antigen retrieval and blocked in PBS with 0.3% Triton X-100 and 5% normal goat serum (NGS) for 10min at room temperature and incubated with primary antibodies diluted in PBS with 5% NGS and 0.1% Triton X-100 at 37˚C for 2h using anti-CD86 antibody (1: 100, Proteintech) and anti-CD163 antibody (1:100, Proteintech). After rinsing three times in PBS with gentle agitation, slices were incubated with the corresponding HRP-conjugated secondary antibody (1: 300, Bioss) diluted in PBS at 37˚C for 30min. Thereafter, the sections were developed with diaminobenzidine for 5min at room temperature and counterstained with hematoxylin. After ehydrated, cleared and mounted, the slides were observed with Olympus Fluorview-500 confocal microscope and quantitative analyzed by Image J software[1].

1. **Adenosine triphosphate (ATP)-ase staining and Oil-Red-O staining**

The transverse serial sections were incubated with calcium chloride solution for 5 min and calcium chloride solution for 30 min. Then, the sections were stained with calcium chloride, cobalt nitrate and ammonium sulphide solutions. Type Ⅰ muscle fiber is light gray or colorless, type Ⅱ muscle fiber is dark gray or black. For Oil-Red-O (ORO) staining to detect lipid deposition, slides were immersed after a washing with PBS for 15 min in the ORO working solution and rinsed with deionized water.

1. **RNA Sequencing and differentially expressed genes analysis**

Total RNA was extracted using the TRIzol reagent according to the manufacturer’s protocol. RNA purity and quantification were evaluated using the NanoDrop 2000 spectrophotometer (Thermo Scientific, USA). RNA integrity was assessed using the Agilent 2100 Bioanalyzer (Agilent Technologies, Santa Clara, CA, USA). Then the libraries were constructed using TruSeq Stranded mRNA LT Sample Prep Kit (Illumina, San Diego, CA, USA) according to the manufacturer’s instructions. The transcriptome sequencing and analysis were conducted by OE Biotech Co., Ltd. (Shanghai, China). The libraries were sequenced on an Illumina HiSeq X Ten platform and 150 bp paired-end reads were generated. Raw data (raw reads) of fastq format were firstly processed using Trimmomatic[5] and the low quality reads were removed to obtain the clean reads. The clean reads were mapped to the human genome (GRCh38) using HISAT2[6]. FPKM[7] of each gene was calculated using Cufflinks[8], and the read counts of each gene were obtained by HTSeq-count[9]. Differential expression analysis was performed using the DESeq (2012) R package[10]. P value < 0.05 and foldchange > 1.5 was set as the threshold for significantly differential expression. Hierarchical cluster analysis of differentially expressed genes (DEGs) was performed to demonstrate the expression pattern of genes in different groups and samples. GO enrichment and KEGG[11] pathway enrichment analysis of DEGs were performed respectively using R based on the hypergeometric distribution.

1. **Flow cytometry**

To determine the percentage of total leukocytes and lymphocyte subsets, blood samples were stained with various monoclonal antibodies (mAbs) and evaluated by flow cytometry. Cells were analyzed on Beckton Dickinson FACS Calibur flow cytometer using flowjo software. The following antibodies were used for flow cytometry analysis: anti-CD4-BV510 100449, Biolegend, San Diego, CA), anti-CD3-PE (100220, Biolegend), anti-CD8a-BV605(100744, Biolegend), anti-CD45-APC (103116, Biolegend), anti-CD19-FITC (115506, Biolegend), anti-CD49b-APC (103516, Biolegend), anti-CD11b-PE (101208, Biolegend), anti-F4/80-BV421 (123137, Biolegend). Data are reported as frequency of live lymphocytes or cells per gram tissue, calculated from frequency of lymphocytes multiplied by absolute count and divided by weight of tissue

1. **Profiling of cytokines/chemokines**

Cytokines and chemokines in paretic gastrocnemius were measured and quantified using the LEGENDplex™ mouse inflammation panel (BioLegend, 740446) according to the manufacturer's instructions. All data were collected on an LSRFortessa and analyzed using LEGENDplex™ software (BioLegend).

1. **Western blot assays**

Muscle tissue was homogenized with an electric homogenizer in RIPA lysis buffer (150mM sodium chloride, 1.0% Triton X-100, 0.5% sodium deoxycholate, 0.1% SDS, 50mM Tris, pH 8.0). Centrifuge for 10min at 12000rpm at 4°C in a microcentrifuge and aspirate the supernatant. Protein concentration was measured with the BCA protein assay kit (CWBIO, China). 30μg of denatured proteins were separated in SDS–PAGE gel (4%-20%, Willget Biotech, China) by electrophoresis under constant voltage (100V in stacking gel for 15min and 150V in revolving gel for 45min), and further transferred onto a PVDF membrane (Millipore, USA). After blocking non-specific binding sites with a 5% BSA for 1 hour at room temperature, the membranes were incubated overnight at 4°C with the appropriate primary antibody diluted in blocking solution: anti-CD86 antibody (1:1000, Proteintech), anti-CD163 (1:1000, Proteintech), anti-TLR4 antibody (1:1000, Santa Cruz), anti-MyD88 (1:1000, Santa Cruz), anti-NFκB (1:1000, Santa Cruz), anti-p-NFκB (1:1000, Santa Cruz) and anti-β-actin (1:5000, Bioss). After three times washes with TBS-T buffer (10mM Tris, 150mM NaCl, 0.05% Tween-20, pH 7.5), the blots were incubated for 2h at room temperature with secondary antibodies: a horseradish peroxidase (HRP)-conjugated goat anti-mouse or rabbit IgG (1/5000, Bioss). The membranes were developed with ECL reagents (CWBIO, China) and imaged via UVP gel imaging system (UVP, USA). Protein band densities were quantified by Image J software (NIH, USA) and were expressed as a percentage of the Sham group[1].

1. **Statistical analysis**

Data are expressed as the mean ± standard error (SEM) of at least three independent experiments.

Statistical tests were done on SPSS 23.0 statistical software (SPSS, Chicago, IL, USA) and GraphPad Prism 9.0 (GraphPad Software Inc., USA). Firstly, a normality test was performed. One-way analysis of variance (ANOVA) for multiple comparisons followed by Tukey’s post hoc test was performed for the data with normal distribution. Kruskal–Wallis test was performed for the data with non-normal distribution. Statistical significance between two groups was determined with unpaired Student’s t test. A probability of 0.05 or less was considered statistically significant.

**References**

[1] Luo L, Li C, Du X, Shi Q, Huang Q, Xu X*, et al.* (2019). Effect of aerobic exercise on BDNF/proBDNF expression in the ischemic hippocampus and depression recovery of rats after stroke. Behav Brain Res, 362:323-331.

[2] Luo L, Li C, Deng Y, Wang Y, Meng P, Wang Q (2019). High-Intensity Interval Training on Neuroplasticity, Balance between Brain-Derived Neurotrophic Factor and Precursor Brain-Derived Neurotrophic Factor in Poststroke Depression Rats. J Stroke Cerebrovasc Dis, 28:672-682.

[3] Zhang Q, Wu JF, Shi QL, Li MY, Wang CJ, Wang X*, et al.* (2019). The Neuronal Activation of Deep Cerebellar Nuclei Is Essential for Environmental Enrichment-Induced Post-Stroke Motor Recovery. Aging Dis, 10:530-543.

[4] Caballero-Garrido E, Pena-Philippides JC, Galochkina Z, Erhardt E, Roitbak T (2017). Characterization of long-term gait deficits in mouse dMCAO, using the CatWalk system. Behav Brain Res, 331:282-296.

[5] Bolger AM, Lohse M, Usadel B (2014). Trimmomatic: a flexible trimmer for Illumina sequence data. Bioinformatics, 30:2114-2120.

[6] Kim D, Langmead B, Salzberg SL (2015). HISAT: a fast spliced aligner with low memory requirements. Nat Methods, 12:357-360.

[7] Roberts A, Trapnell C, Donaghey J, Rinn JL, Pachter L (2011). Improving RNA-Seq expression estimates by correcting for fragment bias. Genome Biol, 12:R22.

[8] Trapnell C, Williams BA, Pertea G, Mortazavi A, Kwan G, van Baren MJ*, et al.* (2010). Transcript assembly and quantification by RNA-Seq reveals unannotated transcripts and isoform switching during cell differentiation. Nat Biotechnol, 28:511-515.

[9] Anders S, Pyl PT, Huber W (2015). HTSeq--a Python framework to work with high-throughput sequencing data. Bioinformatics, 31:166-169.

[10] Love MI, Anders S, Kim V, Huber W (2015). RNA-Seq workflow: gene-level exploratory analysis and differential expression. F1000Res, 4:1070.

[11] Kanehisa M, Araki M, Goto S, Hattori M, Hirakawa M, Itoh M*, et al.* (2008). KEGG for linking genomes to life and the environment. Nucleic Acids Res, 36:D480-484.

**Supplemental Table 1. Descriptive statistics between Sham and MCAO groups.**

| **Variable** | **Sham group** | **MCAO group** | **P value** |
| --- | --- | --- | --- |
| Infarct volume  (% of ipsilateral brain) | 0.8 ± 0.37 | 37.6 ± 4.27 | <0.01 |
| CBF  (% contralateral) | 95.5 ± 1.85 | 25.0 ± 3.24 | <0.01 |
| mNSS | 0.0 ± 0.00 | 9.7 ± 0.61 | <0.01 |

Values are expressed as the mean ± SEM of the mean. Statistical significance was determined with unpaired Student’s t test.

Abbreviation: CBF, cerebral blood flow; mNSS, modified neurological severity score.

**Supplemental Table 2. Descriptive statistics among Sham, MCAO, MOD and HIIT groups.**

| **Variable** | **Sham** | **MCAO** | **MOD** | **HIIT** |
| --- | --- | --- | --- | --- |
| Resting blood lactate concentration | D7: 3.3 ± 0.38  D28: 3.1 ± 0.41 | D7: 5.4 ± 0.41*  D28: 4.9 ± 0.39* | D7: 5.5 ± 0.49*  D28: 4.2 ± 0.40 | D7: 5.6 ± 0.53*  D28: 3.5 ± 0.13^#^ |
| S_LT_ at D7 | 19.5 ± 0.67 | 10.6 ± 1.19** | 9.8 ± 1.12** | 11.1 ± 1.25** |
| Smax at D7 | 27.0 ± 1.16 | 18.5 ± 1.07** | 19.2 ± 1.12** | 17.8 ± 1.16** |
| S_LT_ at D28  (% of D7) | -12.1 ± 3.91 | 18.5 ± 7.97 | 34.7 ± 8.47** | 56.8 ± 9.04**^#^ |
| Smax at D28  (% of D7) | -3.7 ± 3.70 | 8.1 ± 5.40 | 25.0 ± 7.65* | 55.1 ± 8.79**^##+^ |
| Grip strength  (Right forelimb) | 22.8 ± 1.66 | 12.1 ± 0.98** | 19.6 ± 1.10^#^ | 25.5 ± 1.20^##+^ |
| Grip strength  (Left forelimb) | 21.4 ± 0.22 | 15.6 ± 0.64 | 24.2 ± 3.19^#^ | 30.3 ± 0.23*^##^ |
| Grip strength  (All forelimb) | 98.3 ± 5.54 | 62.4 ± 1.99* | 87.3 ± 6.93^#^ | 117.5 ± 9.16^##^ |
| Grip strength  to body weight ratio  (Right forelimb) | 0.8 ± 0.04 | 0.5 ± 0.05** | 0.9 ± 0.05^##^ | 1.1 ± 0.04*^##+^ |
| Grip strength  to body weight ratio  (Left forelimb) | 0.8 ± 0.05 | 0.7 ± 0.01 | 1.1 ± 0.11^##^ | 1.2 ± 0.03**^##^ |
| Grip strength  to body weight ratio  (All forelimb) | 3.8 ± 0.43 | 2.8 ± 0.04 | 3.0 ± 0.19 | 4.8 ± 0.30^##^ |
| Muscle mass | 160.4 ± 4.33 | 131.7 ± 3.81* | 146.2 ± 6.60 | 170.0 ± 7.27^##^ |

**Supplemental Table 2. Continued.**

| **Variable** | **Sham** | **MCAO** | **MOD** | **HIIT** |
| --- | --- | --- | --- | --- |
| Muscle mass to  body weight ratio | 0.7 ± 0.01 | 0.6 ± 0.01* | 0.7 ± 0.02^#^ | 0.7 ± 0.01^##^ |
| Times to fall | 44.2 ± 2.68 | 24.0 ± 2.16* | 44.4 ± 5.52^#^ | 57.8 ± 3.96^##^ |
| Laterality index | 50.8 ± 0.79 | 66.9 ± 3.05** | 58.3 ± 1.67^#^ | 49.8 ± 1.30^##^ |
| Forelimb slip  error percent | 11.7 ± 1.29 | 37.2 ± 3.06** | 19.3 ± 2.40^##^ | 15.5 ± 3.13^##^ |
| Hindlimb slip  error percent | 9.7 ± 2.68 | 14.6 ± 2.45 | 13.0 ± 3.69 | 10.9 ± 1.45 |
| Distance moved | 2270.0 ± 274.80 | 1228.0 ± 102.00* | 2785.0 ± 115.40^##^ | 3286.0 ± 74.03*^##^ |
| Mean velocity | 7.7 ± 0.87 | 4.1 ± 0.34** | 9.4 ± 0.46^##^ | 11.2 ± 0.13**^##^ |
| Stand | RF: 0.16 ± 0.01  RH: 0.17 ± 0.02  LF: 0.16 ± 0.02  LH: 0.17 ± 0.02 | RF: 0.19 ± 0.01  RH: 0.21 ± 0.01  LF: 0.20 ± 0.01  LH: 0.21 ± 0.01 | RF: 0.18 ± 0.01  RH: 0.18 ± 0.01  LF: 0.18 ± 0.01  LH: 0.21 ± 0.02 | RF: 0.17 ± 0.01  RH: 0.16 ± 0.01  LF: 0.17 ± 0.01  LH: 0.18 ± 0.02 |
| Swing | RF: 0.13 ± 0.01  RH: 0.11 ± 0.01  LF: 0.12 ± 0.01  LH: 0.11 ± 0.02 | RF: 0.13 ± 0.00  RH: 0.10 ± 0.00  LF: 0.12 ± 0.00  LH: 0.10 ± 0.01 | RF: 0.13 ± 0.01  RH: 0.11 ± 0.01  LF: 0.13 ± 0.01  LH: 0.10 ± 0.00 | RF: 0.12 ± 0.00  RH: 0.14 ± 0.01  LF: 0.13 ± 0.00  LH: 0.13 ± 0.01 |
| Duty cycle | RF: 56.3 ± 1.43  RH: 60.0 ± 3.67  LF: 56.4 ± 1.98  LH: 59.6± 5.22 | RF: 58.2 ± 1.00  RH: 65.7 ± 1.77  LF: 61.9 ± 0.87  LH: 67.5 ± 0.94 | RF: 57.8 ± 2.29  RH: 61.2 ± 2.73  LF: 59.1 ± 1.58  LH: 65.8 ± 1.96 | RF: 58.4 ± 1.76  RH: 54.0 ± 2.36^#^  LF: 55.5 ± 1.22^#^  LH: 56.6 ± 2.84 |
| Mean Intensity | RF: 71.0 ± 4.34  RH: 82.4 ± 2.24  LF: 71.9 ± 4.50  LH: 79.6 ± 5.21 | RF: 83.2 ± 1.59  RH: 84.3 ± 1.61  LF: 81.5 ± 1.89  LH: 83.3 ± 2.12 | RF: 79.5 ± 4.59  RH: 82.6 ± 2.38  LF: 75.1 ± 4.17  LH: 81.5 ± 4.90 | RF: 73.3 ± 1.73  RH: 72.8 ± 2.45^#+^  LF: 71.2 ± 1.57  LH: 74.7 ± 2.56 |
| Duration | 1.6 ± 0.18 | 1.8 ± 0.10* | 1.8 ± 0.06 | 1.6 ± 0.11^#^ |
| Average Speed | 14.0 ± 1.27 | 12.3 ± 0.40 | 12.4 ± 0.47 | 13.1 ± 0.69 |
| Cadence | 14.0 ± 1.27 | 12.3 ± 0.40* | 12.4 ± 0.47* | 13.1 ± 0.69 |
| CSA of gastrocnemius | 1.6 ± 0.06 | 1.0 ± 0.11* | 1.1 ± 0.12 | 1.6 ± 0.04^#^ |
| Lipid accumulation | 0.6 ± 0.07 | 4.0 ± 0.60* | 3.0 ± 0.48* | 0.9 ± 0.16^#^ |
| Proportion of type I muscle fiber | 55.4 ± 1.48 | 39.9 ± 3.24* | 42.3 ± 2.05* | 50.2 ± 1.67 |
| Proportion of type II muscle fiber | 44.6 ± 1.48 | 60.1 ± 3.24* | 57.7 ± 2.05* | 49.8 ± 1.67 |

**Supplemental Table 2. Continued.**

| **Variable** | **Sham** | **MCAO** | **MOD** | **HIIT** |
| --- | --- | --- | --- | --- |
| IL-1а | 0.13 ± 0.04 | 0.42 ± 0.09 | 0.46 ± 0.11 | 0.25 ± 0.04 |
| IL-1β | 2.44 ± 0.23 | 3.37 ± 0.38 | 3.29 ± 0.24 | 1.80 ± 0.28^#+^ |
| IL-6 | 0.10 ± 0.02 | 0.21 ± 0.02* | 0.13 ± 0.02 | 0.09 ± 0.00^#^ |
| IL-10 | 1.72 ± 0.32 | 0.68 ± 0.13 | 1.07 ± 0.22 | 2.25 ± 0.59^#^ |
| IL-27 | 12.58 ± 1.96 | 9.24 ± 0.92 | 10.18 ± 1.57 | 15.29 ± 2.87 |
| MCP-1 | 0.37 ± 0.14 | 0.66 ± 0.09 | 0.59 ± 0.12 | 0.39 ± 0.12 |
| TNF-α | 0.37 ± 0.06 | 0.58 ± 0.01* | 0.44 ± 0.01 | 0.46 ± 0.03 |
| IFN-γ | 0.40 ± 0.08 | 0.52 ± 0.06 | 0.43 ± 0.04 | 0.42 ± 0.03 |
| Percentage of F4/80+CD11b+ cells in CD45+ cells | 5.9 ± 0.70 | 10.3 ± 1.25* | 81. ± 0.63 | 9.2 ± 2.2 |
| Percentage of CD4+ cells in T cells | 55.3 ± 3.29 | 41.3 ± 3.31 | 54.3 ± 3.33 | 53.3 ± 3.64 |
| Percentage of CD8+ cells in T cells | 35.8 ± 3.88 | 54.5 ± 6.44* | 42.2 ± 3.78 | 35.0 ± 4.94 |
| Ratio of CD4+ and CD8+ cells | 1.8 ± 0.17 | 0.9 ± 0.08** | 1.5 ± 0.21 | 1.7 ± 0.19^#^ |
| Relative level of CD86  (Western blot) | 1.0 ± 0.03 | 1.5 ± 0.10* | 1.3 ± 0.09 | 0.8 ± 0.11^##+^ |
| Relative level of CD163  (Western blot) | 1.0 ± 0.06 | 1.3 ± 0.11 | 1.4 ± 0.08 | 1.8 ± 0.07**^#^ |
| Relative level of TLR4  (Western blot) | 1.0 ± 0.04 | 1.4 ± 0.05** | 1.1 ± 0.03^#^ | 0.9 ± 0.07^##^ |
| Relative level of MyD88  (Western blot) | 1.2 ± 0.13 | 1.7 ± 0.14* | 1.5 ± 0.13 | 0.9 ± 0.09^##+^ |
| Relative level of  NFκB  (Western blot) | 1.0 ± 0.04 | 1.7 ± 0.18* | 1.6 ± 0.18 | 1.2 ± 0.10 |
| Relative level of  p-NFκB  (Western blot) | 1.1 ± 0.10 | 2.6 ± 0.11** | 2.5 ± 0.26** | 1.5 ± 0.07^##++^ |
| Immunoactivity intensity of CD86 | 97.5 ± 8.54 | 165.0 ± 6.46** | 136.5 ± 7.41* | 105.3 ± 10.01^##^ |

**Supplemental Table 2. Continued.**

| **Variable** | **Sham** | **MCAO** | **MOD** | **HIIT** |
| --- | --- | --- | --- | --- |
| Immunoactivity intensity of CD163 | 105.0 ± 7.64 | 90.0 ± 11.55 | 113.3 ± 8.82 | 134.3 ± 8.09^#^ |

Values are expressed as the mean ± SEM of the mean. Statistical significance was determined with one-way analysis of variance (ANOVA) for multiple comparisons followed by Tukey’s post hoc test. **P* < 0.05, ***P* < 0.01 compared with Sham group; ^#^*P* < 0.05, ^##^*P* < 0.01 compared with MCAO group; ^+^*P* < 0.05, ^++^*P* < 0.01 compared with MOD group.

Abbreviation: S_LT_, speed associated with the lactate threshold; Smax, maximal speed; D7, 7 days after cerebral ischemia; D28, 28 days after cerebral ischemia; RF, right forelimb; RH, right hindlimb; LF, left forelimb; LH, left hindlimb. CSA, cross-sectional area.
